# NMR Structure of μ-Conotoxin GIIIC: Leucine 18 Induces Local Repacking of the N-Terminus Resulting in Reduced Na_V_ Channel Potency

**DOI:** 10.3390/molecules23102715

**Published:** 2018-10-22

**Authors:** Peta J. Harvey, Nyoman D. Kurniawan, Rocio K. Finol-Urdaneta, Jeffrey R. McArthur, Dorien Van Lysebetten, Thomas S. Dash, Justine M. Hill, David J. Adams, Thomas Durek, David J. Craik

**Affiliations:** 1 Institute for Molecular Bioscience, The University of Queensland, Brisbane 4072, Australia; peta.harvey@imb.uq.edu.au (P.J.H.); nyoman.kurniawan@cai.uq.edu.au (N.D.K.); Dorien.VanLysebetten@UGent.be (D.V.L.); thomas.dash@imb.uq.edu.au (T.S.D.); justine.hill1@gmail.com (J.M.H.); 2 Illawarra Health and Medical Research Institute, University of Wollongong, Wollongong 2522, Australia; rfinolu@uow.edu.au (R.K.F.-U.); jeffreym@uow.edu.au (J.R.M.); djadams@uow.edu.au (D.J.A.)

**Keywords:** μ-conotoxins, voltage-gated sodium channel blocker, NMR, protein structure

## Abstract

μ-Conotoxins are potent and highly specific peptide blockers of voltage-gated sodium channels. In this study, the solution structure of μ-conotoxin GIIIC was determined using 2D NMR spectroscopy and simulated annealing calculations. Despite high sequence similarity, GIIIC adopts a three-dimensional structure that differs from the previously observed conformation of μ-conotoxins GIIIA and GIIIB due to the presence of a bulky, non-polar leucine residue at position 18. The side chain of L18 is oriented towards the core of the molecule and consequently the N-terminus is re-modeled and located closer to L18. The functional characterization of GIIIC defines it as a canonical μ-conotoxin that displays substantial selectivity towards skeletal muscle sodium channels (Na_V_), albeit with ~2.5-fold lower potency than GIIIA. GIIIC exhibited a lower potency of inhibition of Na_V_1.4 channels, but the same Na_V_ selectivity profile when compared to GIIIA. These observations suggest that single amino acid differences that significantly affect the structure of the peptide do in fact alter its functional properties. Our work highlights the importance of structural factors, beyond the disulfide pattern and electrostatic interactions, in the understanding of the functional properties of bioactive peptides. The latter thus needs to be considered when designing analogues for further applications.

## 1. Introduction

Voltage-gated sodium channels (VGSCs or Na_V_) are responsible for the influx of sodium ions during action potentials in excitable cells [1]. Molecular characterisation of VGSCs has provided considerable insight into their structures, and to date nine subtypes of VGSC α-subunits have been described in mammals (Na_V_1.1–1.9) [2]. μ-Conotoxins present in the venoms of marine cone snails are potent and specific inhibitors of VGSCs and are valuable tools for discriminating between the various channel isoforms [3,4]. μ-Conotoxins are ribosomally-synthesised and post-translationally modified peptides of about 20 amino acids, including six conserved cysteines that stabilise their 3D structure via formation of disulfide crosslink [3]. The μ-conotoxin binding site on VGSCs partially overlaps that of the classical sodium channel inhibitors, tetrodotoxin (TTX) and saxitoxin (STX), and the blocking mechanism of these site 1 toxins appears to involve binding to the outer vestibule and physically occluding the pore [5,6,7,8].

One of the best characterised μ-conotoxins is the 22 amino acid peptide GIIIA from *Conus geographus* [4,9,10,11,12]. This conotoxin potently targets the skeletal muscle subtype Na_V_1.4 (IC_50_: 19 nM), weakly blocks the neuronal subtypes Na_V_1.1, Na_V_1.2 and Na_V_1.6 and does not affect Na_V_1.3, Na_V_1.5, Na_V_1.7 or Na_V_1.8 channels [4]. Mutagenesis studies of GIIIA have shown that cationic residues clustered on one face of the molecule, in particular R13, K16 and R19, make important contributions to the potent blocking efficacy of the toxin [13,14]. This knowledge in conjunction with mutational analysis of the Na_V_1.4 sodium channel has been used to derive a model for μ-conotoxin interaction with the pore and outer vestibule [6,7]. Furthermore, the identified μ-conotoxin–channel interactions have provided insights into the tertiary structure of the sodium channel by revealing that the four internal domains are arranged in a clockwise configuration [15,16].

**Figure 1:**
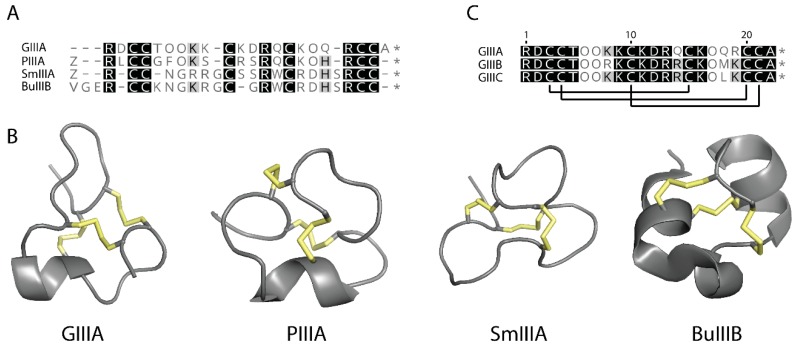
Structural overview of μ-conotoxins. (**A**) Sequence alignment of GIIIA (*C. geographus*), PIIIA (*C. purpurascens*), SmIIIA (*C. stercusmuscarum*) and BuIIIB (*C. bullatus*). Standard one-letter codes are used for amino acids, except for hydroxyproline (O) and pyroglutamate (Z). (**B**) 3D structures of GIIIA (PDB ID: 1TCG) [14], PIIIA (PDB ID: 1R9I) [18], SmIIIA (PDB ID: 1Q2J) [20] and BuIIIB (PDB ID: 2LO9) [21]. Disulfide crosslinks are shown as yellow sticks. (**C**) Sequence alignment of GIIIA, GIIIB and GIIIC. Lines indicate cysteine connectivity. The most significant difference between GIIIC and GIIIA, GIIIB is at position 18 where a leucine residue substitutes for glutamine (GIIIA) and methionine (GIIIB).

μ-Conotoxins with different Na_V_ subtype specificities have been identified from other Conus species, providing additional tools for characterising sodium channel subtypes (Figure 1) [3,4,17]. These toxins possess a number of differences on the primary structure level (Figure 1A) which also affect their three-dimensional structures (Figure 1B). For example, NMR studies demonstrated that PIIIA [18] adopts a significantly different conformation from GIIIA [14,19], presumably to facilitate its interaction with both neuronal and muscle forms of TTX-sensitive VGSCs. Thus, despite the significant conformational constraint afforded by three conserved disulfide bonds, it is possible for these molecules to access different conformations and modulate their ability to recognize and discriminate between different VGSC subtypes.

GIIIA shares considerable sequence homology with other *C. geographus* toxins, particularly with GIIIB and GIIIC as shown in Figure 1C [9,10]. GIIIA and GIIIB have previously been shown to adopt closely related three-dimensional structures [14,19,22]. In the current study, the solution structure of μ-conotoxin GIIIC was determined using NMR spectroscopy to investigate whether the amino acid sequence differences confer any changes in the protein structure. As GIIIC has been shown to demonstrate the highest binding selectivity for rat skeletal muscle vs rat brain VGSCs [18], it was of particular interest to determine whether the presence of a large non-polar leucine residue at position 18 of GIIIC in place of a methionine (GIIIB) or smaller polar residue glutamine (GIIIA) has any effect on the three-dimensional structure. Residues 18 of GIIIA and GIIIB are located at the C-terminus of a small helical region previously shown to be important for μ-conotoxin activity [14]. As leucine residues have a high intrinsic propensity to be located in α-helical elements of secondary structure [23], we hypothesized that leucine substitution may result in elongation of the helix in GIIIC. The NMR-derived structure in fact shows that this does not occur. In contrast, this residue projects into the core of the molecule rather than being surface oriented as in GIIIA and as a consequence the N-terminus is re-modeled and located closer to L18. A comparison of the three-dimensional structures of μ-conotoxins GIIIA, GIIIB and GIIIC reveal important structural differences, including a different conformation of the N-terminus in GIIIC, that may contribute to its high selectivity for the Na_V_1.4 subtype when compared to GIIIA and GIIIB in radioligand displacement studies [18,24].

## 2. Results

### 2.1. Chemical Synthesis 

**Figure 2:**
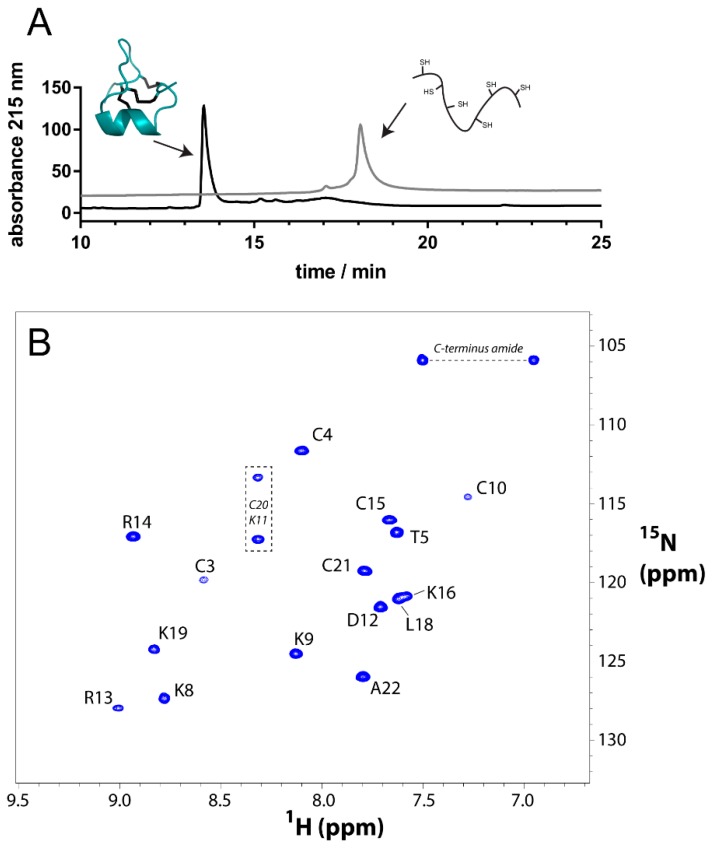
NMR and HPLC analysis of synthetic GIIIC. (**A**) Folding and disulfide formation of GIIIC monitored by RP-HPLC. The grey trace corresponds to the fully reduced polypeptide, whereas the black trace corresponds to the crude folding mixture. (**B**) Natural abundance ^1^H-^15^N HSQC spectrum of synthetic GIIIC. C20 and K11 could not be assigned unambiguously (boxed correlations).

GIIIC and GIIIA were chemically synthesized by automated solid phase peptide synthesis which afforded the crude 22 amino acid residue polypeptides in good yield. Folding and formation of the three disulfide bonds was achieved by incubating the peptides in aqueous ammonium bicarbonate buffer (pH 8.3) for 24 h. Under these conditions, a dominant product eluting earlier than fully reduced polypeptide upon HPLC analysis was formed (Figure 2A) and identified as fully oxidized GIIIC/GIIIA based upon high-resolution MALDI-MS. After purification by RP-HPLC, both peptides were obtained in highly pure form and in good yields. NMR analysis and inspection of the fingerprint region of the ^1^H-^15^N HSQC spectrum of GIIIC indicated excellent dispersion of amide protons suggesting a well-defined fold had been obtained (Figure 2B).

### 2.2. NMR Analysis

The well-dispersed amide protons of μ-conotoxin GIIIC allowed full assignment of backbone resonances using homonuclear 2D NMR methods [25]. Previous analysis of GIIIA [19] and GIIIB [22] reported data acquired at 10 °C since the resonance of Cys10 was extremely broadened at higher temperatures. Whilst this peak is also broadened in GIIIC, as seen in Figure 2, it was indeed clearly visible at 298 K. Since overall amide dispersion of GIIIC was considered optimal at this temperature, NMR data acquired at 298 K were used for the assignment and subsequent structure calculations.

The connectivities of the hydroxyproline residues (Hyp) were not able to be obtained using characteristic Hα-Hδi+1 or Hα-Hαi+1 connectivities due to chemical shift degeneracy [25]. An alternate method of discerning the cis/trans status of the Xaa-Hyp bond, based upon differences between ^13^Cβ and ^13^Cγ chemical shifts, was used [22,26]. For example, since the differences between the ^13^Cβ and ^13^Cγ chemical shifts of Hyp6 and Hyp17 are 33.5 and 33.0 ppm, it was concluded that both the Thr5-Hyp6 and Lys16-Hyp17 peptide bonds are in trans conformations, whereas the smaller difference for Hyp7 of 30.1 ppm indicated that the Hyp6-Hyp7 bond adopts a cis conformation. The assigned ^1^H, ^15^N and ^13^C chemical shifts of GIIIC have been deposited in the BMRB (accession 30519). Comparison of Hα shifts show minimal differences between GIIIA and GIIIC, indicating that their global folds are similar as may be expected based on the high level of sequence similarity.

### 2.3. Three-Dimensional Structure of GIIIC

**Figure 3:**
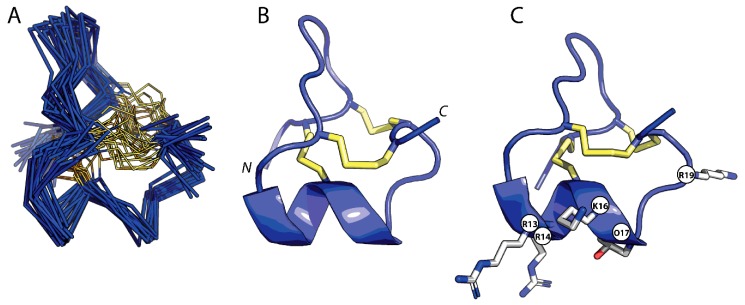
Three-dimensional structure of μ-conotoxin GIIIC. (**A**) Backbone superimposition of the 20 lowest energy structures of GIIIC. (**B**) Cartoon representation of GIIIC (lowest energy structure) and (**C**) highlighting residues important for interaction with VGSCs. The disulfide bonds are shown in yellow.

The three-dimensional structure of GIIIC was calculated using a simulated annealing protocol, based on 179 NOE-derived distance restraints and 33 dihedral restraints (Table 1). Chi1 angle restraints were selected for C3, C10, D12, L18 and C21. Additional restraints included the disulfide connectivities (C3-C15, C4-C20, and C10-C21) and hydrogen bonds as determined from slow D_2_O exchange experiments (C15→D12, K16→R13, and C21→L19). A total of 20 structures were chosen as a representative ensemble for GIIIC, based upon Molprobity scores and lowest total energy [27]. These structures had no NOE violations >0.2 Å and no dihedral angle violations >2°. Geometric and energetic statistics for the calculated NMR structures are shown in Table 1. Figure 3A shows the ensemble of GIIIC structures superimposed over the backbone heavy atoms of residues 1–22. The mean pairwise rmsd is 1.44 ± 0.37 Å and 2.51 ± 0.48 Å for the backbone heavy atoms and all heavy atoms, respectively.

The structure of GIIIC is composed of several turns and a short, single-turn 3_10_-helix from R13 to C15 (Figure 3B,C). Hydrogen bonds present across this helical region are C15→D12 and K16→R13, whilst a third hydrogen bond C21→L19 supports the formation of a type VIII β-turn between K19 and C21 resulting in a small loop at the C-terminus. The three disulfide bonds in GIIIC (C3-C15, C4-C20 and C10-C21) act to stabilize the backbone fold and to present a unique radial orientation of positively charged residues to the solvent. In particular, the N-terminus is tethered to the helix by C3-C15, whilst the C-terminal loop is stabilized by C4-C20 and C10-C21 disulfide bonds.

### 2.4. Pharmacological Activity of GIIIC at VGSCs

**Figure 4:**
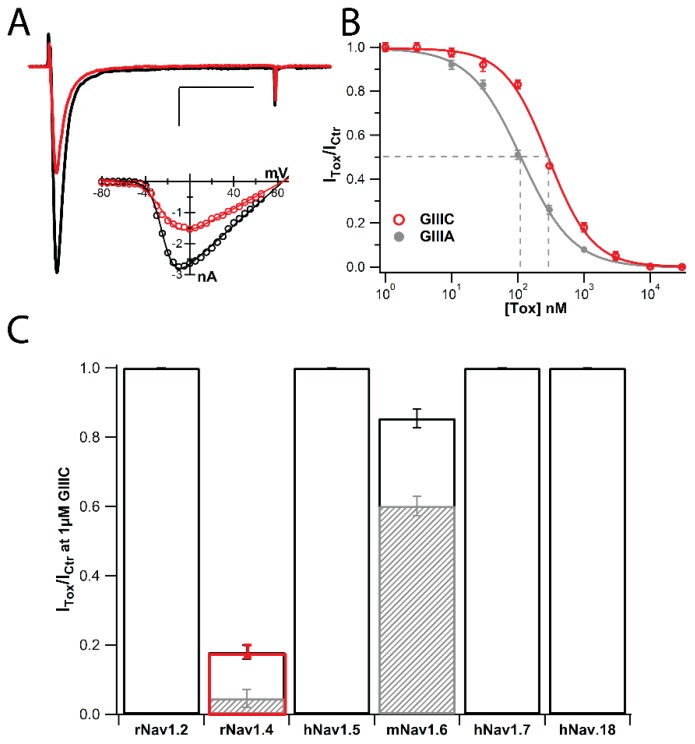
Pharmacological evaluation of GIIIC inhibition of Na_v_ channels. (**A**) Representative current traces of rNa_V_1.4 in control (black) and after 300 nM GIIIC application (red) (holding potential: −120 mV, test potential, −10 mV, 1Hz); Scale bars: 500 pA, 5 ms. Representative I-V relationship shown in the inset. (**B**) Concentration-response curves for GIIIC and GIIIA inhibition of rNa_V_1.4-mediated Na^+^ currents (n = 3–8 for each concentration). (**C**) Fractional block of various Na_V_1.x channel isoforms exposed to 1 μM GIIIC (n = 4–8). Hashed bars correspond to fractional block by GIIIA.

**Figure 5:**
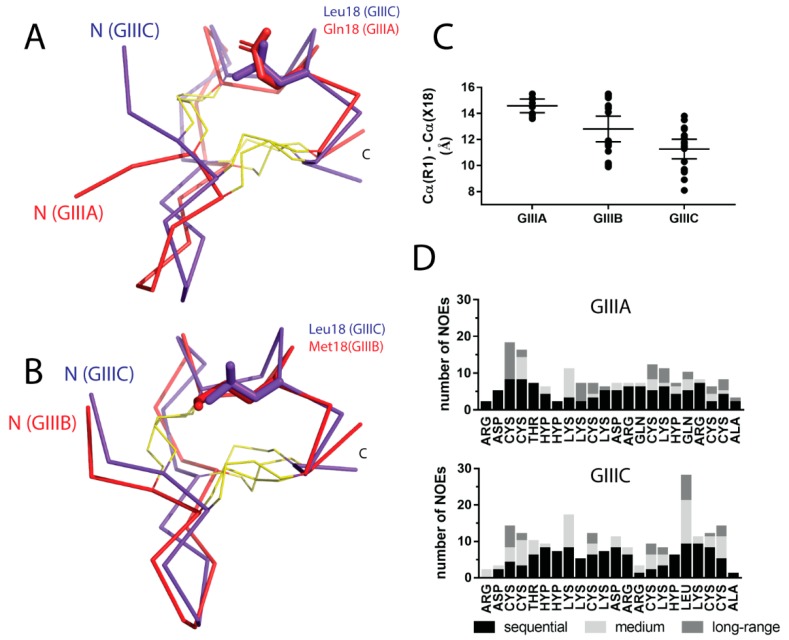
Structural comparison of GIIIC with other *C. geographus* μ-conotoxins. Backbone superimposition of GIIIA (**A**, red) or GIIIB (**B**, red) with GIIIC (blue). Disulfides are shown in yellow and the side chain of residue 18 is indicated in stick representation. (**C**) Distance between Cα atoms of Arg1 and residue 18 in each of the three NMR-derived structures. Data is represented as dot plot (n = 20 for GIIIB and GIIIC, n = 10 for GIIIA) and the mean and 95% confidence intervals are indicated. (**D**) Density of NOE connectivities for GIIIA (upper panel) and GIIIC (lower panel).

In order to establish if the structural differences between GIIIC and GIIIA have functional consequences, the inhibition of Na^+^ currents mediated by mammalian voltage-gated Na_V_1.4 channels was assessed by whole-cell patch clamp electrophysiology of heterologously expressed sodium channels. Representative currents of rNa_V_1.4 in control (black) and in the presence of 300 nM GIIIC (red) demonstrated significant inhibition of Na_V_1.4-mediated currents (Figure 4A). Na^+^ currents elicited by stepping the voltage to −10 mV for 15 ms from a holding potential (Vh) of −120 mV at 0.1 Hz were rapidly and reversibly blocked by 300 nM GIIIC. Current-voltage (I-V) plots in the absence (control) and presence of GIIIC were generated from peak Na^+^ current amplitude during test pulses from −80 to 65 mV (Vh = −120 mV). The inset in Figure 4A corresponds to the same cell and is representative of the data displaying no obvious voltage dependence to current inhibition by GIIIC, consistent with a pore blocking mechanism of inhibition as has been reported for GIIIA block of skeletal muscle Na_V_ channels. We compared the activity of both peptides towards the classical target of GIIIA, rat skeletal muscle, Na_V_1.4. Concentration-response relationships determined for GIIIC and GIIIA (Figure 4B) evidence reduced potency of GIIIC with an IC_50_ of 286 ± 13 nM (Hill coefficient: 1.21 ± 0.07; n = 6) when compared to GIIIA (IC_50_: 110 ± 4 nM, Hill coefficient: 1.11 ± 0.04; n = 5). Given the absolute conservation in the critical charged residues between GIIIA and GIIIC, we propose that the differences in potency we observed may be related to the structural differences between their amino terminal regions (Figure 5).

We also assessed the ability of GIIIC to distinguish among a panel of VGSCs including, Na_V_1.2, Na_V_1.4, Na_V_1.5, Na_V_1.6, Na_V_1.7 and Na_V_1.8. Similar to previous reports of GIIIA inhibition of various VGCS isoforms [4,11,12], GIIIC failed to affect Na^+^ currents mediated by Na_V_1.2, Na_V_1.5, Na_V_1.7 or Na_V_1.8 at a concentration of 1 µM (n = 4, Figure 4C). At the same concentration (1 µM), GIIIC inhibited all but 18 ± 2% (red, n = 7) of the Na_V_1.4 current, whereas GIIIA spared 5 ± 3% (hashed bar, n = 4) of the total current. GIIIA has modest affinity towards Na_V_1.6 channels inhibiting it ~45% at 1 µM (hashed, n = 4), whereas GIIIC inhibited ~14% of Na_V_1.6-mediated currents (n = 5) at the same concentration (Figure 4C). Thus, it seems plausible that the functional properties of GIIIC are a bona fide correlate to its structural idiosyncrasy.

## 3. Discussion

In the present study, we have determined the three-dimensional solution structure of μ-conotoxin GIIIC using 2D 1H NMR spectroscopy. GIIIC adopts a flat ellipsoidal structure composed of several turns, a small N-terminal β-hairpin and a short 3_10_-helix from Arg13 to Cys15. The three conserved disulfide bonds of the μ-conotoxin framework form the core of the molecule and facilitate the projection of numerous charged side chains towards the solvent.

### 3.1. Structural Comparison of GIIIC with Other *μ*-Conotoxins 

The three-dimensional structures of GIIIA (PDB ID: 1TCJ), GIIIB (PDB ID: 1GIB) and GIIIC from *C. geographus* are generally similar, as shown in Figure 5. To make a detailed comparison between these structural ensembles, pairwise fitting was used to calculate backbone rmsd’s of all individual structures within each ensemble and an average obtained. Hence, backbone superimpositions of GIIIA-GIIIC and GIIIB-GIIIC revealed rmsd’s of 2.04 ± 0.25 Å and 1.74 ± 0.33 Å, respectively. Whilst these values are not significantly different, they do suggest that the backbone structure of GIIIC is more similar to GIIIB than GIIIA, which is also represented visually by comparing the lowest energy structures of these peptides (Figure 5A,B). As a result the side chain orientation of residues important for interaction with VGSCs (e.g., Arg13, Arg14, Lys16, Hyp17 and Lys19) is highly similar between GIIIA, GIIIB and GIIIC. The major differences reside in the N-terminus and its orientation in relation to the side chain at position 18. The N-terminus of GIIIC is positioned closer to the core of the molecule, particularly when compared to the N-terminus of GIIIA (Figure 5A). This re-positioning is further illustrated by considering the distance measured between the backbone Cα atoms of Arg1 and residue 18 (GIIIA: Gln; GIIIB: Met; GIIIC: Leu) (Figure 5C), suggesting an overall more compact structure for GIIIC.

In the case of GIIIC, the interaction of L18 with the N-terminus is further substantiated by several NOEs between the Hδ protons of L18 to the NH, Hα and Hβ of C3; and to the NH of C4. These contacts occur as a result of the orientation of the leucine side chain at position 18 towards the core of the molecule and additional support for this is provided by the number of NOE constraints observed for this residue when compared to the corresponding Gln18 in GIIIA (Figure 5D). Notably, the side chain orientation of L18 does not result in any significant changes to the backbone fold of GIIIC, that is the alignment of disulfide bonds or positioning of the small helix, as this residue fits neatly into the core.

The side chain orientations of L18 in GIIIC and M18 in GIIIB are similar (Figure 5B), although the conformations of their N-termini differ and the sidechain of M18 appears to be more exposed and solvent accessible. Methionine to leucine substitutions in T4 lysozyme have been shown to either increase or decrease molecular stability depending on their location in the structure [28]. Substitutions of methionine to leucine at fully buried sites decreased T4 lysozyme stability, most likely due to steric hindrance, whereas substitutions at partially or fully exposed sites were better tolerated because of less residual strain. In the latter case, however, the increase in protein stability would not be as high as expected because of unfavorable solvent exposure and unoptimized solvent transfer potential. In GIIIC, the orientation of L18 towards the core of the molecule might result in decreased solvent accessibility and increased tethering of the N-terminus. This position is favored as steric hindrance is low and the exposure to the solvent is minimized.

The current structural analysis of GIIIC comes a considerable time after that of GIIIA or GIIIB and it was important to check that structural differences between these peptides were not the result of differences in conditions of sample preparation or data acquisition. Thus, all samples were prepared in aqueous solvent under similar conditions of pH and concentration. We do note that the current analysis was based on data acquired at 298 K rather than the slightly lower temperature reported for GIIIA and GIIIB. We considered that 298 K is more biologically relevant and the similar number of inter-residue distance restraints used in each of the structures suggests that temperature differences do not affect the three-dimensional solution structures.

The structural differences between GIIIA, GIIIB and GIIIC are unexpected given that these peptides share the same disulfide framework and have considerable sequence homology. In most cases, proteins with a conserved disulfide framework adopt a similar structure and any differences in their biological activity and/or specificity likely arises primarily from differences in the composition of residues in the solvent-exposed loops. In addition, cis/trans isomerisation about a single bond in PIIIA was shown to alter the shape of part of the cysteine framework [18]. GIIIA, GIIIB and GIIIC differ by only four residues at positions 8, 14, 18 and 19 (Figure 1C). Substitution between lysine, arginine or glutamine at positions 8, 14 and 19 are fairly conservative changes in regards to charge or size of these sidechains; however, position 18 is unique in that there is substitution of an uncharged glutamine to a hydrophobic residue, either methionine in GIIIB or leucine in GIIIC. Furthermore, in this study we have shown that the seemingly minor M18L substitution transmits a significant conformational change to the N-terminus of GIIIC. These observations demonstrate that the μ-conotoxin framework has some conformational flexibility that may affect the interaction between these toxins and their target ion channels.

### 3.2. Implications for *μ*-Conotoxin Interaction with Sodium Channels 

Insights into the architecture of the pore and outer vestibule of the Na_V_1.4 channel have been obtained using the three-dimensional structures of GIIIA and GIIIB as molecular calipers [6,15,16]. Important residues for the activity of the μ-conotoxins, R13, Q14 (R14 in GIIIB), K16, O17 and R19 [13,29,30], are clustered on the helical face of the molecule (Figure 3C). This region has been suggested to make direct interactions with the channel pore, whereas D12, Q14, O17 and K16 have been described as forming a collar above and around R13, which extends towards the selectivity filter [15]. R1 of GIIIA has also been shown to be of moderate to high importance in μ-conotoxin binding, however, the current models of μ-conotoxin-channel interaction differ over its role [15,16]. Most studies have focused on the electrostatic interactions between charged amino acids in μ-conotoxins and their Na_V_ channel counterparts; hence peptide positions occupied by non-polar residues have been neglected. GIIIC effectively constitutes a natural variant to assess the role of the non-polar amino acid at position 18. The structural constraints imposed by substitution of Gln (in GIIIA) by Leu (in GIIIC) at position 18 would affect the interactions of critical N-terminal charged residues R1 and D2 [15,16,29] with the channel pore and thus provide a molecular basis to GIIIC’s relative lower potency against the skeletal muscle NaV channel. Finally, the functional properties of GIIIC coupled to the structure determined in this study are useful to probe and further refine the emerging models of the Na_V_1.4 channel pore.

## 4. Materials and Methods

### 4.1. Peptide Synthesis 

GIIIA and GIIIC were prepared by automated Fmoc solid phase peptide synthesis on Rink amide resin. The side chain protecting groups used were Arg(Pbf), Asp(OtBu), Cys(Trt), Hyp(tBu), Lys(Boc) and Thr(tBu). Following chain assembly, peptides were side-chain deprotected and cleaved from the solid support using a cleavage cocktail of trifluoroacetic acid (TFA):triisopropylsilane:H_2_O (95:2.5:2.5) (*v/v/v*). After stirring for 1.5 h at room temperature, the majority of the TFA was evaporated under vacuum and the peptide was precipitated with ice-cold diethyl ether. Crude peptide was dissolved in 50% acetonitrile (ACN)/water containing 0.05% TFA and lyophilized. The target peptide was purified by preparative RP-HPLC on a Phenomenex C_18_ column using linear gradients of solvent B (ACN:H_2_O:TFA; 89.5:10:0.05) in solvent A (H_2_O:TFA; 99.5:0.05). Peptide purity and identity were assessed by ESI-MS on a API-2000 mass spectrometer (Applied Biosystems, Foster City, CA, USA) and by analytical scale μHPLC on a Nexera system (Shimadzu, Melbourne, VIC, Australia) equipped with an Agilent Zorbax C_18_ column (1.8 μm, 2.1 × 100 mm). Fractions containing the desired product were pooled, lyophilized and stored at −20 °C. The reduced peptide was oxidized at room temperature for 24 h in 0.1 M ammonium bicarbonate buffer (pH 8.3) at a peptide concentration of 0.3 mM and the final product was purified by preparative RP-HPLC as described above. High-resolution MALDI-MS (α−cyano-4-hydroxycinnamic acid as a matrix): M_found_ 2592.22 Da; M_calc_ (most abundant isotope composition) 2592.23 Da.

### 4.2. NMR Spectroscopy 

The NMR samples were prepared in 90% H_2_O/10% D_2_O or 100% D_2_O at peptide concentrations of 2 mM and pH of 3.5. NMR spectra were recorded on an Avance III 600 MHz spectrometer (Bruker, Sydney, NSW, Australia) equipped with a cryogenically cooled probe at temperatures ranging from 283–303 K. 2D NMR experiments included TOCSY with an 80 ms MLEV-17 spin lock [31,32], NOESY [33] with a mixing time of 200 ms, both ^1^H-^15^N and ^1^H-^13^C HSQC [34], and E-COSY [35]. Solvent suppression was achieved using an excitation sculpting sequence [36]. Slowly exchanging amide protons were detected by acquiring a series of 1D and TOCSY spectra immediately following dissolution of the fully protonated peptide in D_2_O. Whilst D_2_O exchange was monitored for 24 h, slow exchangers were defined as those amide protons remaining beyond 2 h. Spectra were referenced to internal 4,4-dimethyl-4-silapentane-1-sulfonate (DSS). All spectra were processed with Topspin 3.5 (Bruker, Sydney, NSW, Australia) and analyzed using CCPNMR analysis [37].

### 4.3. Structure Calculations 

Distance information was obtained from the NOESY spectra at 298 K in both 10% and 100% D_2_O, using CYANA’s calibration tools to convert peak volumes into upper distance restraints. Preliminary structures were generated using CYANA based upon these distance restraints and disulfide bond restraints. Hydrogen bond restraints were then included, as indicated by slow D_2_O exchange and sensitivity of amide proton chemical shift to temperature, along with backbone dihedral angle restraints generated using TALOS+ [38]. Several side chain χ1 angles were restrained based on the strength of observed intra-residue Hα-Hβ and NH-Hβ NOEs and ^3^JHα-Hβ coupling patterns determined from the E-COSY. A final set of structures was generated within CNS [39] using torsion angle dynamics, refinement and energy minimization in explicit solvent, and protocols as developed for the RECOORD database [40]. Final structures were assessed for stereochemical quality using MolProbity [27]. The assigned ^1^H, ^15^N and ^13^C chemical shifts of GIIIC have been deposited in the BMRB (accession 30519) and structural coordinates have been deposited in the PDB (6MJD). 

### 4.4. Pharmacology

Plasmids carrying the coding sequence of rat (r)Na_V_1.2, rNa_V_1.4, human (h)Na_V_1.5, mouse (m)Na_V_1.6, hNa_V_1.7 and hNa_V_1.8 were transiently transfected with Lipofectamine 2000 (Invitrogen, Melbourne, VIC, Australia) into TSA201 cells. Depolarization-activated sodium channel currents were recorded 18–24 h later at room temperature by the whole-cell configuration of the patch-clamp technique using a Multiclamp 700B patch clamp amplifier (Molecular Devices, Sunnyvale, CA, USA) coupled to a Digidata 1550 and Clampex 10 acquisition system. The bath solution contained (in mM): 142.5 NaCl, 5 KCl, 2 CaCl_2_, 1 MgCl_2_, 10 HEPES, and 10 D-glucose (pH 7.4). The pipette electrodes had resistance of 1–3 MΩ when filled with internal solution composed of (in mM): 140 CsF, 1 MgCl_2_, 10 HEPES, and 1 EGTA (pH 7.2). Membrane currents were acquired at 25 kHz and online low pass filtered at 10 kHz. Data is expressed as mean ± SEM.

## Figures and Tables

**Table 1:** Statistical analysis of GIIIC structures ^a^.

Experimental Restraints	
total no. distance restraints	179
intraresidue	71
sequential	59
medium range, *i* − *j* < 5	35
long range, *i* − *j* ≥ 5	14
hydrogen bond restraints	6
dihedral angle restraints	
phi	17
psi chi1	11 5
Deviations from idealized geometry	
bond lengths (Å)	0.010 ± 0.001
bond angles (deg)	1.294 ± 0.046
impropers (deg)	1.38 ± 0.18
NOE (Å)	0.014 ± 0.002
cDih (deg)	0.126 ± 0.132
Mean energies (kcal/mol)	
overall	−664 ± 42
bonds	9.3 ± 1.0
angles	34.0 ± 2.7
improper	10.7 ± 2.1
van Der Waals	−56.6 ± 5.7
NOE	0.04 ± 0.01
cDih	0.13 ± 0.18
electrostatic	−760 ± 43
Violations	
NOE violations exceeding 0.2 Å	0
dihedral violations exceeding 2.0 Å	0
Rms deviation from mean structure, Å	
backbone atoms	1.44 ± 0.37
all heavy atoms	2.51 ± 0.48
Stereochemical quality ^b^	
residues in most favoured Ramachandran region, %	96.6 ± 4.1
Ramachandran outliers, %	0.2 ± 1.1
unfavourable sidechain rotamers, %	0.0 ± 0.0
clashscore, all atoms	6.5 ± 3.0
overall MolProbity score	1.5 ± 0.2

^a^ All statistics are given as mean ± SD; ^b^ According to MolProbity [27].
